# Fecal Microbiota Transplantation Ameliorates Experimentally Induced Colitis in Mice by Upregulating AhR

**DOI:** 10.3389/fmicb.2018.01921

**Published:** 2018-08-24

**Authors:** Yan-Ling Wei, Yu-Qin Chen, Hao Gong, Ning Li, Kang-Qi Wu, Wang Hu, Bin Wang, Kai-Jun Liu, Liang-Zhi Wen, Xiao Xiao, Dong-Feng Chen

**Affiliations:** Department of Gastroenterology, Institute of Surgery Research, Daping Hospital affiliated to the Army Medical University, Chongqing, China

**Keywords:** fecal microbiota transplantation, aryl hydrocarbon receptor, dextran sulfate sodium, IL-10, TGF-β

## Abstract

Ulcerative colitis (UC) is a chronic non-specific inflammatory disease that occurs in the colon and rectum. While fecal microbiota transplantation (FMT) is gaining attention as a clinical treatment of UC, the molecular mechanisms behind this effect have yet to be fully understood. A C57BL/6 mouse model was established to test whether FMT promotes the recovery of colon inflammation. Administration of 2% dextran sulfate sodium (DSS) for 7 days successfully induced acute colitis, as evidenced by diarrhea, hematochezia and colon shortening as well as a decrease in body weight. FMT alleviated the severity of colon mucosa injury and improved histological alterations compared with that of the DSS group. In addition, FMT promoted homeostasis of the intestinal microbiota. Furthermore, FMT upregulated the expression of aryl hydrocarbon receptor (AHR), interleukin-10 (IL-10), and transforming growth factor beta (TGF-β) in colon tissues. These results suggest that the significant anti-inflammatory effect of FMT may be attributed to its promotion of IL-10 and TGF-β production and AHR activation. Based on these results, FMT had a favorable therapeutic effect on DSS-induced colitis.

## Introduction

Ulcerative colitis (UC) is a chronic non-specific inflammatory disease of unknown cause. Because UC is refractory and often recurs, it has become a common challenge for gastroenterologists and greatly compromises patients’ quality of life (Shi et al., 2016). While the incidence of UC is lower in China than in some developed countries, its incidence in China has been on the rise in the past 30 years (Rooks and Garrett, 2016). The pathogenesis of UC is not fully understood and is thought to be caused by a variety of factors, such as genetic predisposition, environmental factors, eating disorders, emotional distress, immune disorders, and microbiota (Qin et al., 2010; Goodrich et al., 2014; Ji et al., 2016). In recent years, aided by the rapid development of high-throughput DNA sequencing and bioinformatics, it has been thought that impaired homeostasis and functional alterations of intestinal microbiota and their metabolites play important roles in the pathogenesis of UC (Round and Mazmanian, 2009; Wang et al., 2014; Vindigni et al., 2016; Sartor and Wu, 2017). Therefore, restoring and maintaining the homeostasis of intestinal microbiota and their metabolites has become a viable therapeutic option for UC.

Fecal microbiota transplantation (FMT) is a procedure in which large amounts of intestinal microbiota from prescreened healthy donors are transplanted to the gastrointestinal tract of recipients to help redress intestinal dysbacteriosis in patients and reconstitute functional intestinal microecology (Borody and Campbell, 2012; de Groot et al., 2017). Previous studies have demonstrated that FMT has achieved favorable results in the treatment of clostridium difficile infection (CDI) and inflammatory bowel disease (IBD) (Cammarota et al., 2014; Wang et al., 2016; Ma et al., 2017). Nevertheless, it remains unclear how FMT functions as an alternative treatment for restoring the balance of intestinal microbiota.

Aryl hydrocarbon receptor (AhR) is an important ligand-dependent transcriptional regulator in the cytoplasm. It is a member of the Bhlh-PAS subfamily of the helical-loop-helix (HLH) superfamily (Fujii-Kuriyama et al., 1994; Denison and Nagy, 2003). AhR was first thought to mediate the pathological effects of dioxins and other toxic contaminants, while AhR regulation by endogenous metabolites is considered to play an important role in essential biological processes such as inflammation regulation, immunity, cell differentiation and apoptosis (Singh et al., 2011; Safe et al., 2013; Hubbard et al., 2015; Villa et al., 2016). Studies have shown that AhR can associate with the intestinal metabolite tryptophan and its downstream metabolites such as indole-3-acetic acid (IAA); the resulting complex is translocated into the nucleus, where AhR is activated and regulates proliferation and differentiation of intestinal inflammatory cells and the transcription and expression of inflammation-related factors (Singh et al., 2011; Takamura et al., 2011; Lanis et al., 2017; Schiering et al., 2017). In addition, Takamura et al. (2011) found that *Lactobacillus bulgaricus* OLL1181 alleviated colitis induced by dextran sodium sulfate (DSS) in mice by activating the AhR signaling pathway. However, it remains to be confirmed whether intestinal microbiota and bacterial metabolites that help maintain intestinal homeostasis can protect the intestinal tract after the transplantation of fecal bacteria by regulating the AhR signaling pathway to suppress inflammation in UC. Using a mouse model of DSS-induced intestinal inflammation, we observed the changes in intestinal microbiota and metabolites, and the expression of AhR, interleukin-10 (IL-10), and transforming growth factor beta (TGF-β) after FMT. We examined the specific mechanism by which restoration of intestinal microbiota protects the intestinal tract against inflammation and provide insights into FMT-based treatments for UC.

## Materials and Methods

### Reagents

Dextran sodium sulfate (MP Biomedicals, Solon, OH, United States) was stored at room temperature and was dissolved in distilled drinking water to a concentration of 2% before delivery to the mice.

### Animals and Induction of Colitis

Female, 7-week-old C57BL/6 mice were purchased from the Experimental Animal Center, Institute of Field Surgery, Daping Hospital. Mice were randomly assigned to the following groups (*n* = 10 per group): control, DSS alone, and DSS plus FMT (DSS+FMT). Control mice were fed distilled water daily. Mice in the DSS group were treated daily with 2% DSS for 7 days, followed by distilled water (without DSS) for 7 days. Mice in the DSS plus FMT group were treated daily with 2% DSS for 7 days and then fed daily with FMT for the next 7 days by gastric lavage.

### Fecal Microbiota Transplantation (FMT)

Fresh murine fecal samples were collected directly from the rectums of 10 age- and sex-matched control mice. The collection was always performed at 8 o’clock in the morning, mixed with sterile normal saline, and then homogenized immediately. Homogenates were then passed through a 20 μm pore nylon filter to remove large particulate and fibrous matter. The filters were centifuged at 6000 *g* for 5 min at 4°C using a SorvallSS-34 centrifuge. The underlayer precipitate was dissolved in normal saline to a concentration of 400 mg/mL and used for transplantation (100 g/50 kg per mouse). The supernatant (500 μl) was stored at -80°C until it was used for analysis of microflora.

Fecal microbiota transplantation was performed for every mouse once a day. Mice were anesthetized using chloral hydrate administered by intraperitoneal injection. Intracolonic injection was performed carefully though a catheter made from a round-tip silicone tube with a diameter of 1 mm. The tube was inserted 3.5 cm into the mouse colon. After injection, the mouse was held vertically with its head down for 1 min to prevent loss of the injectant.

### Clinical Evaluation and Disease Activity Index

Clinical scores of colitis, including weight change, diarrhea, colorectal bleeding and survival, were monitored every day. Overall disease severity was assessed by a clinical scoring system, and a disease activity index (DAI) score was calculated for each animal (Zhang et al., 2014).

### Histological Analysis

Colon length was measured and then colon tissues taken approximately 2 cm from the anal end were fixed in 10% formalin and embedded in paraffin. Ten-micrometer serial sections were obtained for hematoxylin and eosin (HE) staining to observe pathological changes in the colon.

### Transmission Electron Microscopy

Colon tissues taken approximately 0.5 cm from the anal end were dissected, rinsed with PBS, and fixed in electro mirror fixation (Olympus, Japan). Samples were dehydrated using increasing concentrations of ethanol, then infiltrated and embedded in epoxy resin. One-micrometer sections were prepared to select representative areas by light microscopy, and 80 nm ultrathin sections were then cut using a diamond knife. Sections were mounted on 200 mesh copper grids and stained with 4% aqueous uranyl acetate and aluminum citrate. Sections were observed with a JEOL-1400PLUS transmission electron microscope (JEOL, Japan).

### Microbiota Analysis by 16S rRNA Sequencing

Mouse stools were collected daily by stimulating the anus and stored at -80°C. The stools and FMT bacterium fluid were sent together to Shenzhen PUYUAN for 16S rRNA sequencing by an Illumina miseq platform. The structure and quantity of the microbiota were analyzed by PCA analysis and cluster analysis.

### High-Performance Liquid Chromatography – Mass Spectrometry (HPLC-MS) Analysis

Metabolites were extracted from the colon tissue or fecal pellets using a methanol/chloroform extraction method. Cold methanol/chloroform (2:1, v/v; 1.5 ml) was added to a colon or fecal sample and homogenized on ice. The sample tube was centrifuged at 15000 *g* for 10 min at 4°C, and the supernatant was transferred to a new sample tube through a 70-μm cell strainer. Ice-cold water (0.6 ml) was added, and the sample tube was vortexed and centrifuged (15000 *g*, 5 min, 4°C) to obtain phase separation. The upper phase was separated and collected in fresh sample tubes then stored at -80°C. Then, the samples were sent to Army Medical University for detection and analysis using Analyst software (version 5; Agilent Technologies, Foster City, CA, United States).

### Immunohistochemistry (IHC) Analysis

Dissected pieces of colon were immersed and fixed overnight at 4°C in 10% paraformaldehyde before being embedded in paraffin. Unstained 4 μm sections were cut, deparaffinized and dehydrated using increasing concentrations of ethanol, sealed with 3% H_2_O_2_ at room temperature, high pressure-repaired with citrate buffer solution (pH = 6.0), and resealed with goat serum. Then, sections were incubated in a 1:200 dilution of anti-AhR rabbit polyclonal IgG (Affinity Bioscience, Cincinnati, OH, United States), a 1:300 dilution of anti-IL-10 mouse monoclonal IgG (Abcam, Cambridge, MA, United States) and a 1:200 dilution of anti-TGF-β rabbit polyclonal IgG (Abcam, Cambridge, MA, United States) overnight at 4°C. Next, sections were washed in TBST and incubated with biotinylated goat anti-mouse-rabbit IgG (Abcam, Cambridge, MA, United States) for 1 h. The staining was developed using the DAKO Liquid DAB+substrate chromogen system (Dako, Carpinteria, CA, United States) for 8 min. Slides were washed in water, counterstained with hematoxylin and dehydrated before mounting. Slides were examined using light microscopy with an Olympus IX51 microscope.

### Western Blot Analysis

Colon tissues were milled in ice-cold RAPI (Beyotime, China) containing 2 mM phenylmethylsulfonyl fluoride (PMSF). The homogenates were then centrifuged at 12000 *g* for 10 min at 4°C. The supernatant was collected, and the protein concentration was determined by a BCA assay kit (Beyotime, China). Samples were separated by 8% SDS–PAGE gels and then transferred onto PVDF membranes. Blotted membranes were blocked with 5% skim milk and then incubated with primary antibody against AhR (1:1000, rabbit anti-AhR, Affinity, Sterling, VA, United States), IL-10 (1:1000, mouse anti-IL-10; Abcam, Cambridge, MA, United States), TGF-β (1:1000, rabbit anti-TGF-β, Abcam, Cambridge, MA, United States), or β-actin (1:1000, mouse anti-β-actin; Beyotime, China). Next, the membranes were incubated with horseradish peroxidase-conjugated secondary antibody (ZSGB-Bio, China). The specific reaction was visualized by a chemiluminescent substrate (ECL). The mean density of each band was analyzed using Quantity One analysis software. Results were expressed as AhR/β-actin, IL-10/β-actin, or TGF-β/β-actin. Each point was repeated in triplicate.

### RNA Extraction and RT-qPCR

RNA was isolated using the SYBR Select Master Mix Kit according to the manufacturer’s instructions. cDNA was synthesized with Superscript II (Invitrogen). RT-qPCR was performed using SYBR Green reagent (Invitrogen, refer to the safety section in the SYBR Select Master Mix protocol) and gene-specific primers (IL-10-F:CGGGAAGACAATAACTGCACCC, IL-10-R: CGGTTAGCAGTATGTTGTCCAGC, TGF-β -F:TGATACGCCTGAGTGGCTGTCT, TGF-β-R:CACAAGAGCAGTGAGCGCTGAA). Reactions were performed using an Abi Prism 7500 (Applied Biosystems, Foster City, CA, United States) thermal cycler. Housekeeping genes were carefully selected for each experiment so that their expression levels did not exhibit significant differences between treatments. Relative expression was calculated using the formula 2-aact. The average from the controls was taken as 1, and fold change for each treatment was calculated accordingly. Each sample was tested in triplicate for qPCR.

### Immunofluorescence

Distal colons were excised from all mice, extensively flushed with phosphate-buffered saline (PBS), embedded into optimal cutting temperature compound, and flash frozen. Embedded colon samples were stored at -80°C. Sections (8 μm) were post-fixed with paraformaldehyde (4°C for 15 min), air-dried, and incubated in 5% serum (specific serum targeting host of the secondary antibody) and 2% bovine serum albumin for 30 min before incubating overnight with primary antibody for AhR (1:100) or Foxp3 (1:200) for 8 h at 4°C. Sections were washed and incubated with rhodamine-conjugated secondary antibody (1:200) or FITC (1:200) for 1 h at room temperature. All sections were counter-stained with DAPI (5 min, 1:10,000) to identify cell nuclei.

### Statistical Analysis

All statistical analyses were performed using the SPSS19.0 software package (Chicago, IL, United States). Data were expressed as the mean ± standard deviation (SD). Differences between individual groups were first compared using analysis of variance (one-way ANOVA) or two-tailed *t*-Test. All reported *p*-values were two-sided, and a value of *p* at 0.05 was considered statistically significant.

## Results

### FMT Alleviated DSS-Induced Weight Loss in Mice

**FIGURE 1:**
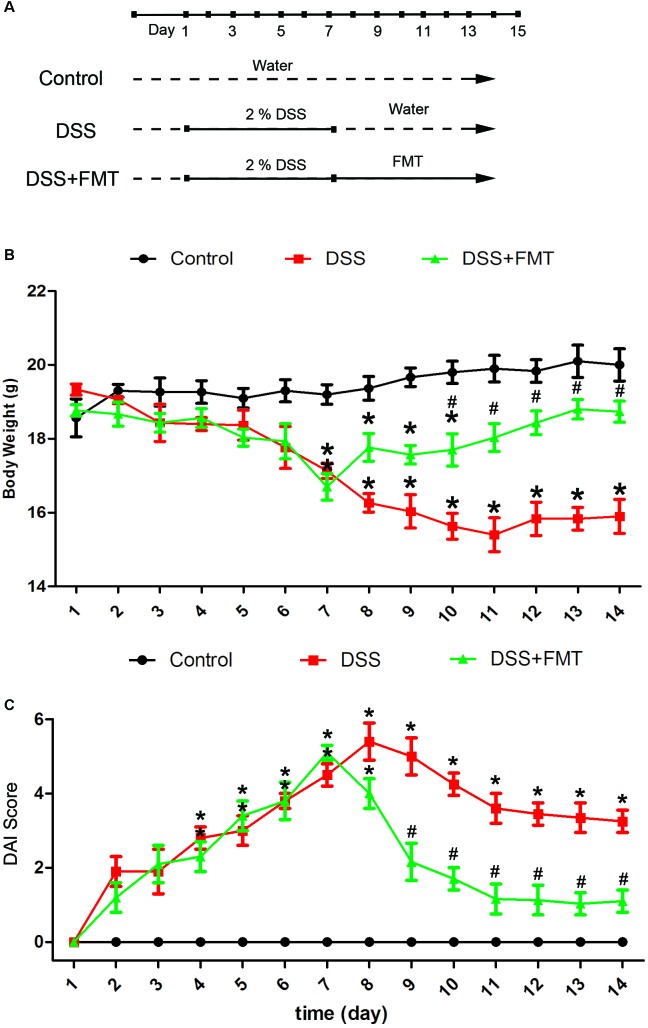
Fecal microbiota transplantation reversed body weight loss and decreased the Disease Activity Index (DAI) score of mice with acute colitis induced by DSS. **(A)** Experimental protocol. **(B)** Average body weight at the indicated time point in each group. **(C)** Several clinical indexes of disease, including weight loss, stool consistency, occult blood positivity, and gross rectal bleeding, were monitored to calculate a cumulative DAI score. DAI scores were recorded at the indicated time point in each mouse. Data are shown as the mean ± SD (*n* = 3 mice per group) of triplicate experiments. ^∗^*p* < 0.05 compared to the control group. ^#^*p* < 0.05 compared to the DSS group.

Mice were observed daily. We found that DSS caused weakness, anorexia, huddling, and lack of vitality, whereas control mice had a normal diet and activity. As shown in **Figure 1**, compared with that of the control group, the weight of mice exposed to DSS decreased to a minimum at day 8, after which body weight did not change noticeably. By comparison, the weight of mice in the FMT group decreased until day 7 before recovering slowly. At day 7, significant weight loss was noted in the DSS group and the DSS plus FMT group compared with that of the control group (*P* < 0.05). From day 10 to day 14, the DSS group experienced a significant decline in body weight compared with that of the control group (*P* < 0.05), and there was no significant difference in body weight between the DSS plus FMT group and the control group (*P* > 0.05). However, the body weight of the DSS plus FMT group was significantly higher than that of the DSS group (*P* < 0.05; **Figure 1**).

### FMT Alleviated DSS-Induced Disease Activity

After DSS exposure, disease activity in mice in the DSS group and the DSS plus FMT group increased over time compared with that in the control group, peaking at days 8 and 7, respectively. Thereafter, disease activity was alleviated in both the DSS group and the DSS plus FMT group. At day 7, a significant increase in the DAI was noted in the DSS group and the DSS plus FMT group compared with that in the control group (*P* < 0.05). From day 9 to day 14, the DSS group had significantly higher DAI scores than those in the control group (*P* < 0.05), and there was no significant difference in DAI scores between the DSS plus FMT group and the control group (*P*> 0.05). However, the average DAI score of the DSS plus FMT group was significantly lower than that of the DSS group (*P* < 0.05; **Figure 1**).

### FMT Promoted Recovery of Colon Length in Mice After DSS Exposure

**FIGURE 2:**
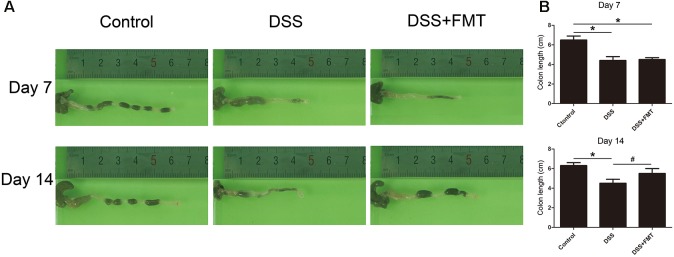
Fecal microbiota transplantation prevented DSS-induced shortening of colon length. Colon length in each group was measured at day 7 and day 14 of the experiment. **(A)** Representative colon images at the indicated time point in each group. **(B)** Data are shown as the mean ± SD (*n* = 3 mice per group). ^∗^*p* < 0.05 compared to the control group. ^#^*p* < 0.05 compared to the DSS group.

To investigate the effect of DSS and FMT on colon length in mice, we measured colon length in mice at days 7 and 14. At day 7, the colon lengths in the DSS group and the DSS plus FMT group were significantly shorter than were those in the control group (*P*< 0.05). Colon length at day 14 in the DSS group did not differ markedly from that at day 7 but was significantly shorter than that of the control group. However, colon length at day 14 in the DSS plus FMT group was longer than that at day 7 and was not significantly different from that of the control group (*P* < 0.05; **Figure 2**).

### FMT Promoted Repair of DSS-Induced Colonic Histological Damage and Mucosal Epithelial Ultrastructure

**FIGURE 3:**
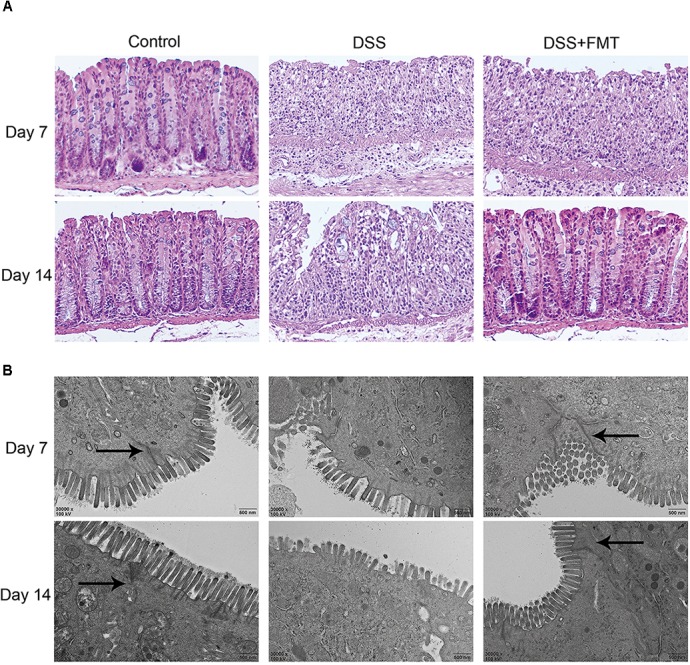
Fecal microbiota transplantation promoted the repair of DSS-induced histological damage to colon tissue. **(A)** Colon tissue sections were stained with HE. Scale bars = 50 μm. **(B)** FMT promoted the repair of microscopic damage in colon tissue induced by DSS. The colon tissue sections of mice in each group was measured by transmission electron microscopy. The arrow represents tight connection. Scale bars = 500 nm. Representative histology from the control, DSS, and DSS+FMT groups.

The intestinal mucosa of mice in each group were stained with HE to observe histological damage and ultrastructural changes of the intestinal mucosa epithelium under transmission electron microscopy. As shown in **Figure 3**, in the control group, the colon surface appeared smooth and covered with a slight amount of mucus. Blood vessels were visible under the mucosa, and no ulcer was formed. In mice exposed to DSS, the colon became congested and edematous and was accompanied by ulceration. HE staining showed that in the control group the colon epithelium was intact, the glands were arranged regularly, capillaries and a few lymphocytes were clearly seen in the lamina propria, and there was no obvious infiltration of inflammatory cells. At day 7, in the DSS group and the DSS plus FMT group, the mucosal epithelium became discontinuous, glands were disarrayed, crypts became shortened, and the mucosa and the submucosa were infiltrated by many inflammatory cells. At day 14, colon injury remained largely unrepaired in the DSS group, while colonic congestion and edema were largely resolved in the DSS plus FMT group, with arrangement of the glands in a more regular pattern and milder inflammatory cell infiltration (**Figure 3A** and **Supplementary Figure S1**).

At day 7, in the colonic mucosal epithelial cells of the DSS group and the DSS plus FMT group, the mitochondria became swollen, and autophagy occurred in some cells. Intestinal epithelial microvilli became fewer and were broken, and the length of the colon was shortened. At day 14, there was no obvious recovery of the intestinal mucosal epithelium and microvilli in the DSS group. The microvilli of colonic epithelial cells increased significantly and became densely arranged and longer after FMT treatment (**Figure 3B**).

### FMT Improved DSS-Induced Imbalances of Intestinal Microbiota and Metabolites

**FIGURE 4:**
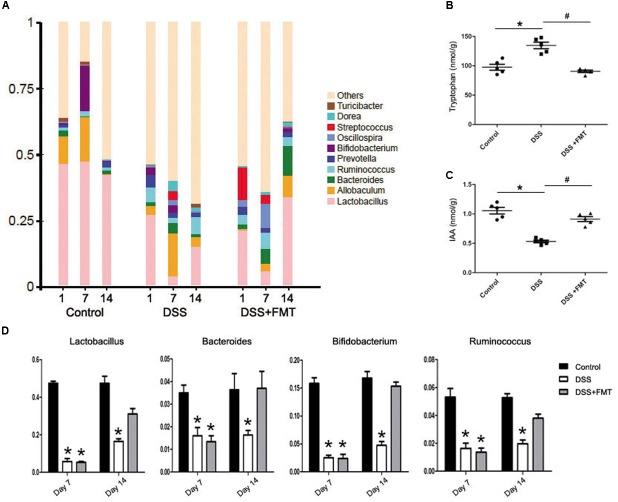
Fecal microbiota transplantation relieved the intestinal flora disturbance of mouse colon induced by DSS. **(A)** Histogram representing bacterial taxonomy based on 16S rRNA gene sequencing at the genus level for colon fecal samples. **(B, C)** Tryptophan-derived bacterial metabolites in intestinal contents of mice force-fed with DSS. **(D)** Intestinal flora data are shown as the mean ± SD (*n* = 6 per group). ^∗^*p* < 0.05 compared to the control group. ^#^*p* < 0.05 compared to the DSS group.

16S rRNA analysis of intestinal microbiota was performed on feces of each group of mice on days 1, 7, and 14. The amounts of *Lactobacillus, Bacteroides, Bifidobacterium*, and *Ruminococcus* bacteria at day 1 in the DSS group and the DSS plus FMT group were decreased compared with those in the control group. At day 7, the amounts of *Lactobacillus, Bacteroides, Bifidobacterium*, and *Ruminococcus* bacteria were decreased significantly in the DSS group and the DSS plus FMT group (*P*< 0.05), while differences in the amounts of other strains of bacteria were not statistically significant. The amounts of *Lactobacillus, Bacteroides*, and *Bifidobacterium* bacteria at day 14 in the DSS group were increased compared with those at day 7, but were still lower than those in the control group. However, the amounts of *Lactobacillus* and *Bifidobacterium* bacteria at day 14 in the FMT group almost recovered to the control level, and the amounts of other strains of bacteria were significantly reduced compared with those at day 7 and had returned to the control level (*P*< 0.05; **Figure 4A**).

Tryptophan and IAA levels in intestinal metabolites were determined by high-performance liquid chromatography the tryptophan levels at day 14 in the DSS group were significantly increased compared with those in the control group (*P*< 0.05), but there was no remarkable difference in tryptophan levels between the FMT group and the control group. However, tryptophan levels at day 14 in the FMT group were significantly lower than those in the DSS group (*P*< 0.05). Meanwhile, IAA levels in the DSS group were decreased significantly compared with those in the control group, while there was no significant difference in IAA levels between the FMT group and the control group. Additionally, IAA levels in the FMT group were significantly higher than those in the DSS group (*P*< 0.05; **Figures 4B,C**).

### FMT Promoted AhR Expression in Colon Tissue of DSS-Exposed Mice

**FIGURE 5:**
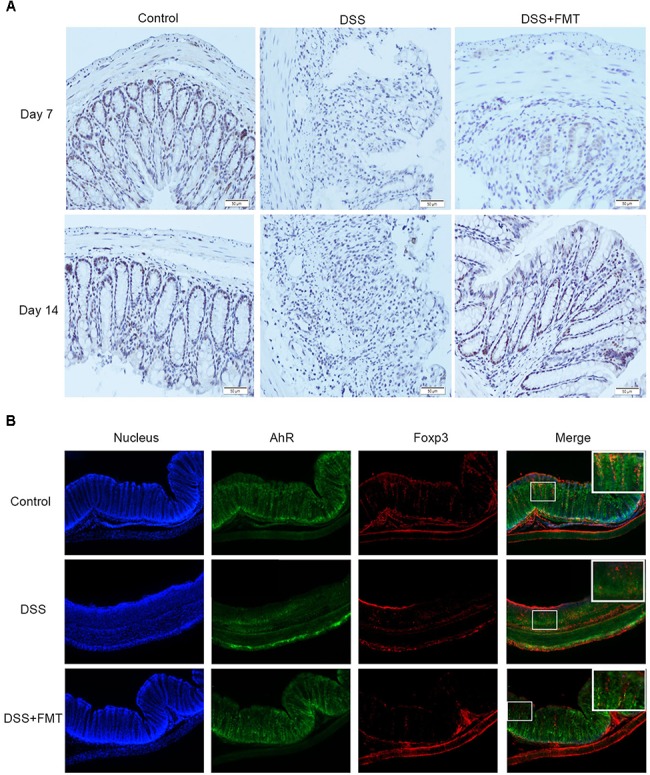
Compared with that in the control group, decreased AHR expression in colon tissues of mice was induced by DSS and detected by immunohistochemistry **(A)**, Representative colon images at the indicated time point in each group. Scale bars = 50 μm. Representative example of an immunofluorescence double staining of Foxp3 (Red) and AhR (Green) in colon tissues **(B)**.

Immunohistochemical staining was used to detect the expression of AhR in colon tissue. After mice were exposed to DSS, the expression of AhR at day 7 in the DSS group and the FMT group was significantly lower than that in the control group (*P* < 0.05). The expression of AhR at day 14 in the DSS group was not significantly different from that at day 7 but was significantly lower than that in the control group (*P* < 0.05). The expression of AhR at day 14 in the FMT group was significantly higher than that at day 7 (*P* < 0.05) but was not significantly different from that in the control group (**Figure 5A**).

Immunofluorescence staining was used to test the expression of AhR in Treg cells. As shown in **Figure 5B**, compared to the control group, the DSS group exhibited significantly decreased expression of AhR in Treg cells. However, the expression of AhR in the DSS plus FMT group was not significantly different from the control group (**Figure 5B**).

### FMT Promoted IL-10 and TGF-β Expression in Colon Tissue of DSS-Exposed Mice

**FIGURE 6:**
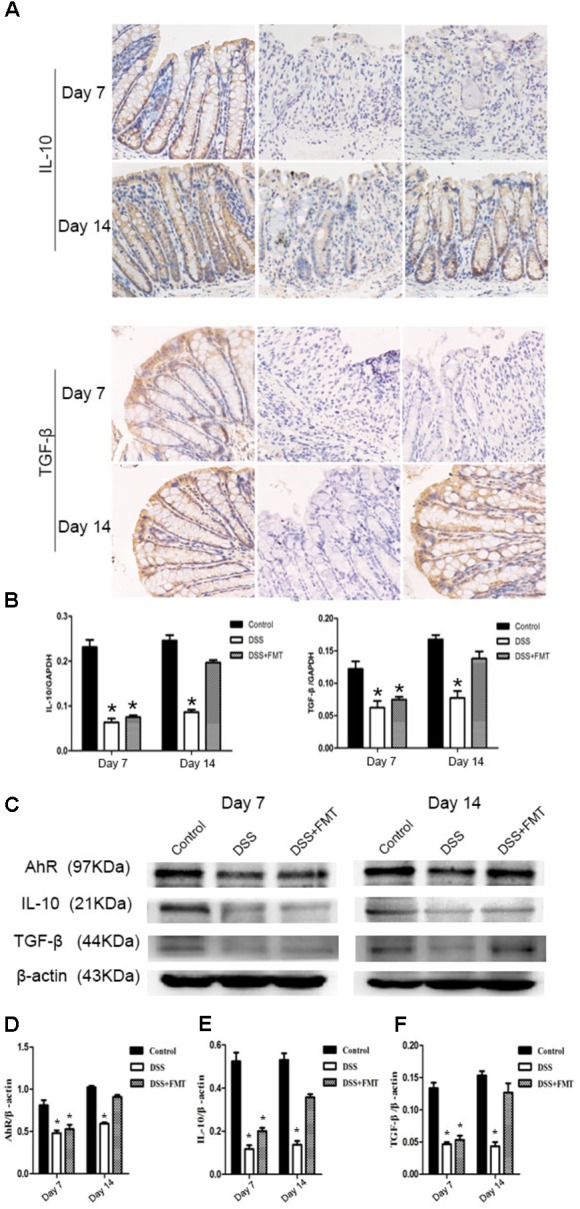
Compared with that in the control group, decreased IL-10 and TGF-β expression in colon tissues of mice was induced by DSS and detected by immunohistochemistry. Representative photographs of immunohistochemical staining of IL-10 and TGF-β at the indicated time point in each group **(A)**. Scale bars = 50 μm. **(B)** The mRNA level of IL-10 and TGF-β in colon tissues of different groups. **(C)** Protein expression of AhR, IL-10, and TGF-β in colon tissues of different groups. **(D)** The ratio of AhR/β-actin in each group. **(E)** The ratio of IL-10/β-actin in each group. **(F)** The ratio of TGF-β/β-actin in each group. ^∗^*p* < 0.05 compared to the control group.

Immunohistochemical staining revealed that IL-10 and TGF-β were expressed in the colon tissues of all groups of mice. After mice were exposed to DSS, the expression levels of IL-10 and TGF-β at day 7 in the DSS group and the FMT group were significantly lower than those in the control group (*P* < 0.05). The expression levels of IL-10 and TGF-β at day 14 in the DSS group were not significantly different from those at day 7 but were significantly lower than those in the control group (*P*< 0.05). The expression levels of IL-10 and TGF-β at day 14 in the FMT group were significantly higher than those at day 7 (*P* < 0.05) but not significantly different from those in the control group. Meanwhile, Western blot and RT-PCR were also used to determine the expression of IL-10 and TGF-β. These results were similar to the immunohistochemical results (**Figure 6**).

## Discussion

The pathogenesis of UC is very complex, as the imbalance of intestinal microbial homeostasis, in addition to genetic, environmental and immune factors, plays an important role in the development of UC (Frank et al., 2007; Morgan et al., 2012; Baumler and Sperandio, 2016). In recent years, mouse models have been used to study the interaction between host and microorganism in the intestinal microbiome, enhancing the understanding of the pathological mechanism of UC as well as the screening of new drug targets (Gkouskou et al., 2014). DSS can cause inflammatory damage to intestinal mucosa, perhaps by causing DNA damage, thus inhibiting the repair of epithelial cells and activating inflammatory cells (Mizoguchi, 2012). Extensive T cell reaction and the secretion of a large number of inflammatory factors, such as IL-6 (Interleukin-6) and TNF-α (Tumor necrosis factor-α) were observed after the induction of DSS in the colon of mice (Strober et al., 2002). The pathological changes in the mouse model of acute colon inflammation induced by DSS were similar to those in humans, and this model has been widely used in the study of the pathological mechanism of UC. Therefore, in the present study, we aimed to investigate the effects of FMT on microbial and metabolite imbalance in mice with DSS-induced colitis and examined changes in the AhR signaling pathway and its downstream factors by observing changes in the general condition of mice before and after FMT as well as the macroscopic and microscopic changes in the colon.

In our study, we successfully established a mouse model of UC by feeding 2% DSS to C57BL/6 mice for 7 days. During model establishment, the mice showed progressively decreased activity, diarrhea, hematochezia, and weight loss. At the same time, we also observed the colon mucosa congestion and edema, colon wall thickness and hardened lumen, absence of crypt structure, decreased epithelial and goblet cells, infiltration of inflammatory cells and shortened colon length. After FMT, transmission electron microscopy showed disappearance of colonic mucosal ulcers, only a little erosion and slight infiltration of inflammatory cells, crypt structure integrity, and increased number and length of microvilli of colon epithelial cells. These results suggest that the DSS-induced colon inflammation and tissue damage of mice are significantly reduced after FMT treatment, and FMT therapy significantly relieved the symptoms of DSS-induced colitis in mice. These results agree with the research of Tian et al., 2016, which illustrated that FMT can markedly relieve inflammation of the colon and promote the recovery of its organizational structure, thus promoting colon function of colon in mice, which may also be related to gradual increases in the weight of mice after FMT. Thus, our results suggest FMT can promote the recovery of colon tissue damage and reduced the severity of colon inflammation in mice, supporting an anti-inflammatory role of FMT.

The interaction between the intestinal microbiome, intestinal metabolites and host immunity plays an important role in the development of the host intestinal immune system, and even the maintenance of intestinal function. In normal lumen, pro-inflammatory and anti-inflammatory factors are balanced; however, in the lumen of UC, many mast cells and T cells are activated and produce inflammatory factors while anti-inflammatory factors are relatively decreased, causing colon tissue damage (Biasi et al., 2013; Osaka et al., 2017; Weingarden and Vaughn, 2017). Recent research has confirmed that the reduction of anti-inflammatory factors (such as IL-4, IL-10, IL-13, and TGF-β) was closely related to the pathogenesis of UC (Xiong et al., 2013); at the same time, the downstream response caused by anti-inflammatory factors in the process of inflammation can promote the recovery from inflammation (Lanis et al., 2017). With the continuous development of disease molecular medicine, some nuclear transcription factor signaling pathways, such as the AhR pathway, have attracted more attention from researchers, especially the secretion and regulation of inflammatory and anti-inflammatory cytokines associated with the activation of the AhR signaling pathway (Honda and Littman, 2012). It has been reported that AhR participates in a variety of biological activities, such as colon immune regulation and intestinal metabolism. The activation of AhR in the intestinal tract was related to levels of tryptophan and its metabolites in the intestinal tract (Hubbard et al., 2015; Lanis et al., 2017). Microbiota and their metabolites in the intestinal tract can activate the AhR pathway in the form of ligand dependence, then AhR regulation of the differentiation of immune cells promotes or regulates the release of anti-inflammatory factors (Maynard et al., 2012; Bessede et al., 2014; Villa et al., 2016; Kawajiri and Fujii-Kuriyama, 2017). Therefore, AhR is a key regulatory factor in intestinal immunity. To verify whether FMT can affect the expression of AhR, we examined the bacteria and bacterial metabolites in mice feces as well as the expression of AhR in Treg cells in the colon. 16S rRNA gene sequencing analysis found that the number of *Lactobacillus, Bacteroides, Bifidobacterium, and Ruminococcus* were increased in the FMT treatment group, and chromatography showed the levels of intestinal tryptophan and IAA were nearly recovered to the control level. These results were essentially identical with our clinical data in patients. Some intestinal flora showed tryptophan metabolism activity, which can metabolize tryptophan into IAA (Zelante et al., 2013). Therefore, intestinal Lactobacillus and Bacteroides increased after FMT treatment, strengthening the metabolism of tryptophan to IAA, the increased levels of which can activate the cytoplasmic AhR receptors and the high activity of AhR is related to the release of anti-inflammatory factors (Bessede et al., 2014; Hanieh, 2014). Many studies have also shown that tryptophan regulates expression of AhR in mice with UC, which is consistent with our results (Takamura et al., 2011; Schiering et al., 2017). Previous studies have shown that IL-10 plays an important role in the maintenance of intestinal homeostasis, and the absence of IL-10/IL-10R function in mice can cause inflammation of the colon. The expression of IL-10 in intestinal epithelial cells plays an important role in intestinal epithelial defense and barrier function (Kominsky et al., 2014; Lanis et al., 2017). Additionally, TGF-β can regulate a variety of innate and adaptive immunity processes (Blois et al., 2014). In our study, we observed increased expression of IL-10 and TGF-β after FMT treatment. These results indicated that increased anti-inflammation factors may be a reason FMT promotes the recovery of colitis induced by DSS. Based on these results, a possible mechanism is that FMT recovers intestinal flora diversity, promoting intestinal structure and function and maintaining intestinal flora and their metabolites (tryptophan, IAA). Then, endogenous IAA combines with AHR and intestinal AHR ligands promote AHR transport into the nuclei of Treg cells, promoting release of anti-inflammatory factors, thus helping to alleviate the inflammation of the colitis induced by DSS in mice. Although we have no direct evidence of tryptophan, IAA and AHR regulating T cell differentiation and anti-inflammatory factor release, some scholars have confirmed that AHR activation can promote gathering of Treg cells and release of IL-10 and TGF-β (Oh-Oka et al., 2017).

**FIGURE 7:**
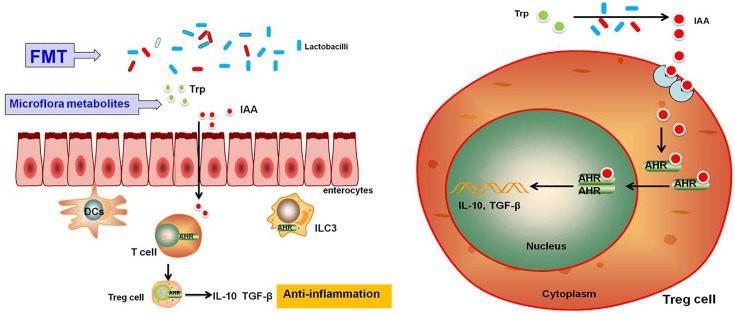
Schematic representation of the effects of FMT on DSS-induced ulcerative colitis in mice through maintenance of the composition of intestinal microbiota and overview of the signaling pathways activated downstream of AHR. Upon treatment with FMT, homeostasis of the intestinal microbiota and metabolites will be restored, resulting in the activation of the AhR signaling pathway followed by AhR promotion of Treg cell differentiation, which is accompanied by the induction of expression of anti-inflammatory cytokines such as IL-10 and TGF-β.

In summary, our results suggest that FMT can effectively relieve clinical symptoms and pathological changes in the mouse model of acute colitis induced by DSS. Its anti-inflammatory mechanism may be that FMT maintains the steady-state and activates AHR, promoting release of anti-inflammatory factors (IL-10, TGF-β); however, the exact mechanism will be the subject of future research (**Figure 7**).

Fully understanding the function and composition of bacteria and host interactions will help us find a deeper understanding of the pathogenesis of UC and help make targeted clinical interventions and treatments. Our results showed that FMT regulates intestinal dysbacteriosis, namely, restoring intestinal flora diversity and steady-state, and may become a kind of natural treatment strategy for UC. However, further basic research and clinical trials are needed to understand the very large role of the microbiota on maintaining human intestinal homeostasis.

## Conclusion

In summary, our results demonstrated that FMT, compared with the control fecal sample, can alleviate acute colitis induced by DSS in mice. In addition, FMT can promote homeostasis of the intestinal microbiota and their metabolites such as AHR ligands (tryptophan and IAA). Our results also suggested that FMT upregulates the expression of AHR, IL-10 and TGF-β in colon tissues. Thus, we propose that FMT could increase the secretion of anti-inflammatory cytokines by activating AhR signaling in Treg cells of colon tissues, which are responsible for FMT-reduced colon inflammation.

## Ethics Statement

This study was carried out in accordance with the recommen dations of “Daping Hospital Animal Experiment Center Guidelines, Animal Ethics Committee of Daping Hospital affiliated to Army Medical University.” The protocol was approved by the “The Animal Experiment Ethics Committee of Army Medical University,” and the ID was 20160801.

## Author Contributions

Y-LW designed the experiments and wrote and revised the draft manuscript and subsequent manuscripts. Y-QC, NL, K-QW, and HG performed the experiments, participated in the design and coordination of the experiments, and helped to draft and revise the manuscript. WH participated in the FMT procedure. BW, K-JL, and L-ZW participated in the analysis of data and drafted the manuscript. XX and D-FC supervised and coordinated the project. All authors read and approved the final manuscript.

## Conflict of Interest Statement

The authors declare that the research was conducted in the absence of any commercial or financial relationships that could be construed as a potential conflict of interest.

## Supplementary Material

FIGURE S1 Colon tissue sections were stained with HE (4×).
