# Supplementary figures and images for: Fecal Microbiota Transplantation Ameliorates Experimentally Induced Colitis in Mice by Upregulating AhR
Source: Front Microbiol. 2018 Aug 24;9:1921. doi: 10.3389/fmicb.2018.01921 (PMC6118168; doi:10.3389/fmicb.2018.01921)

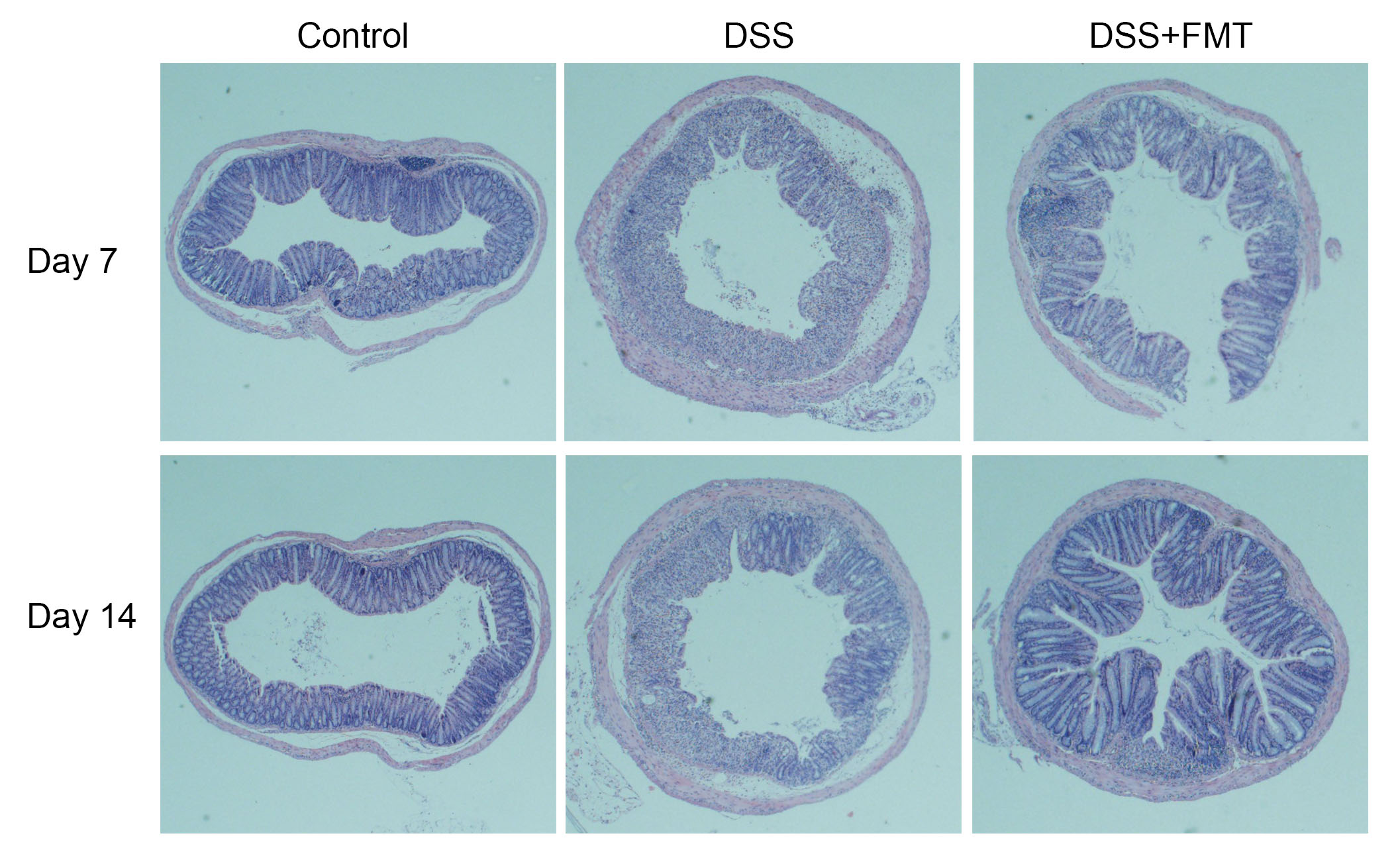

Supplement: FIGURE S1 — Colon tissue sections were stained with HE (4×). [file Image_1.JPEG]
